# Photodynamic Therapy with 5-Aminolevulinic acid (ALA) Impairs Tumor Initiating and Chemo-Resistance Property in Head and Neck Cancer-Derived Cancer Stem Cells

**DOI:** 10.1371/journal.pone.0087129

**Published:** 2014-01-24

**Authors:** Chuan-Hang Yu, Cheng-Chia Yu

**Affiliations:** 1 School of Dentistry, Chung Shan Medical University, Taichung, Taiwan; 2 Department of Dentistry, Chung Shan Medical University Hospital, Taichung, Taiwan; 3 Institute of Oral Sciences, Chung Shan Medical University, Taichung, Taiwan; National Cancer Institute, United States of America

## Abstract

**Background:**

Head and neck cancer (HNC) ranks the fourth leading malignancy and cancer death in male population in Taiwan. Despite recent therapeutic advances, the prognosis for HNC patients is still dismal. New strategies are urgently needed to improve the chemosensitization to conventional chemotherapeutic drugs and clinical responses of HNC patients. Studies have demonstrated that topical 5-aminolevulinic acid-mediated photodynamic therapy (ALA-PDT) is being used in the treatment of various human premalignant and malignant lesions with some encouraging clinical outcomes. However, the molecular mechanisms of ALA-PDT in the therapeutic effect in HNC tumorigenesis and whether ALA-PDT as chemosensitizer for HNC treatment remain unclear. Accumulating data support cancer stem cells (CSCs) contributes chemo-resistance in HNC. Based on the previous studies, the purpose of the study is to investigate the effect of ALA-PDT on CSCs and chemosensitization property in HNC.

**Methodology/Principal Finding:**

CSCs marker ALDH1 activity of HNC cells with ALA-PDT treatment as assessed by the Aldefluor assay flow cytometry analysis. Secondary Sphere-forming self-renewal, stemness markers expression, and invasiveness of HNC-CSCs with ALA-PDT treatment were presented. We observed that the treatment of ALA-PDT significantly down-regulated the ALDH1 activity and CD44 positivity of HNC-CSCs. Moreover, ALA-PDT reduced self-renewal property and stemness signatures expression (Oct4 and Nanog) in sphere-forming HNC-CSCs. ALA-PDT sensitized highly tumorigenic HNC-CSCs to conventional chemotherapies. Lastly, synergistic effect of ALA-PDT and Cisplatin treatment attenuated invasiveness/colongenicity property in HNC-CSCs.

**Conclusion/Significance:**

Our results provide insights into the clinical prospect of ALA-PDT as a potential chemo-adjuvant therapy against head and neck cancer through eliminating CSCs property.

## Introduction

Head and neck cancer (HNC) is one of the most common cancers in the world and one of causes of cancer-related death due to frequent recurrence after chemotherapy resistance [1]. Despite improvements in the diagnosis and management of HNC, long-term survival rates have improved only marginally over the past decade [1]. New drugs or strategies are urgently needed to improve the chemosensitization to conventional chemotherapeutic drugs and clinical responses of HNC patients [2].

Recent studies have pointed out resistance to conventional radiotherapies/chemotherapies treatment is a major clinical criteria for characterizing cancer stem cells (CSCs) with self-renewal and highly tumorigenenic capacity in malignant tumors including HNC[2]–[4] Both intracellular ALDH1+ and CD44+ cells have been proposed to exhibit CSCs properties and have been used as CSCs functional markers for head and neck cancer-derived cancer stem cells (HNC-CSCs)[5]–[7]. Nevertheless, searching an effective approach targeting HNC-CSCs to improve HNC -related malignancies is urgently required [7].

Photodynamic therapy (PDT) involves two individually non-toxic components, light and photosensitizer [8]. PDT is a new treatment and holds considerable promise for many solid tumors[9]. Studies have demonstrated that topical 5-aminolevulinic acid-mediated PDT (ALA-PDT) is being used in the treatment of various human premalignant and malignant lesions with some encouraging clinical outcomes [10]–[13]. PDT might have the potential of inhibiting the metastasis of incompletely treated HNC [14]. In a mice Lewis lung carcinoma model, ALA-PDT decreased the metastasis of cancer cells in vivo[15]. In addition, ALA-PDT increases apoptotic ability of oral cancer cells through NF-κB/JNK signaling [16]. ALA-PDT also abrogated migration capacity of oral cancer cells by down-regulation of FAK and ERK [17].

Based on the previous studies, the purpose of the study is to investigate the effect of ALA-PDT on chemosensitization and CSCs property in HNC. Overall, we first demonstrated ALA-PDT effectively reduce CSC-like property including ALDH1 activity, CD44 positivity, self-renewal, invasion, and enhanced the chemosensitivity in HNC. ALA-PDT would be a potential chemo-adjuvant therapy and may be beneficial for anti-CSCs treatment in HNC.

## Materials and Methods

### Primary cultivated cells from HNC tissues

Surgical tissue specimens from HNC patients were collected after obtaining written informed consent and this study was approved by The Institutional Review Board in Chung Shan Medical University Hospital (CSMUH No: CS10249). Primary HNC cells were established as previously described [6], [18]. In brief, after surgical removal of the HNC tissues, the tissues were washed 3 times in glucose containing HBSS, then the samples were sliced at a thickness of 300 µm and the sliced tissues were immersed in 0.1% (w/w) collagenase containing glucose containing HBSS for 15 minutes at 37°C and subjected to rotation shaker shaking at 125 rpm. HNC primary culture were cultured in DMEM (Invitrogen, Carlsbad, CA, USA) with 10% fetal bovine serum, supplemented with 1 mM sodium pyruvate, non-essential amino acids, 2 mM L-glutamine, 100 units/mL penicillin, and 100 µg/mL streptomycin under standard culture conditions (37°C, 95% humidified air, 5% CO_2_).

### Chemicals

5-ALA was obtained purchased from Sigma (St. Louis, MO, USA). Just before use, 5-ALA was further diluted in culture medium to appropriate final concentrations.

### HNC cell lines cultivation and 5-ALA-based photodynamic therapy

For ALA-PDT studies, the cells were incubated with 5-ALA for 3 hours, and then irradiated with red light of 635±5 nm at various doses.

### Enrichment of sphere-forming HNC cancer stem cells (HNC-CSCs)

Spheres from HNC cells were isolated in medium consisting of serum-free DMEM/F12 medium (GIBCO), N2 supplement (GIBCO), 10 ng/mL human recombinant basic fibroblast growth factor (basic FGF), and 10 ng/mL epidermal growth factor (EGF) (R&D Systems, Minneapolis, MN). Cells were plated at a density of 10^4^ live cells/10-mm low attachment dishes, and the medium was changed every other day until the tumor sphere formation was observed in about 1 week [2].

### Aldefluor assay

Aldefluor assay kit is purchased from StemCell Technologies, Inc. (Vancouver, BC, Canada) 1×10^5^ cells will be suspended in 50 µl of assay buffer and added Aldefluor to final concentration of 1 µM. For ALDH1 inhibitor control, DEAB will be added to final concentration of 150 µM. Cells will be then incubated at 37°C for 45 min and stained with 7-AAD on ice for further 5 min. After washed with PBS, green fluorescence positive cells in live cells (7AAD-) will be analyzed by flow cytometry (FACSCalibur™, BD Bioscience) by comparing the fluorescence intensity of DEAB treated sample and these cells will be represented as cells with high ALDH activity (ALDH+ cells)[2].

### SYBR Real-time reverse transcription-polymerase chain reaction (RT-PCR)

Total RNA of cells was purified using Trizol reagent (Invitrogen, Carlsbad, CA, USA) according to the manufacturer's protocol. Briefly, the total RNA (1 µg) of each sample was reversely transcribed by Superscript II RT (Invitrogen, Carlsbad, CA, USA). Then, the amplification was carried out in a total volume of 20 µl containing 0.5 µM of each primer, 4 mM MgCl_2_, 2 µl LightCycler™ –FastStart DNA Master SYBR green I (Roche Molecular Systems, Alameda, CA, USA) and 2 µl of 1∶10 diluted cDNA. The *GAPDH* housekeeping gene was amplified as a reference standard. *GAPDH* primers were designed: *GAPDH* (forward): GGGCCAAAAGGGTCATCATC (nt 414–434, GenBank accession no. BC059110.1), *GAPDH* (reverse): ATGACCTTGCCCACAGCCTT (nt 713–733). PCR reactions were prepared in duplicate and heated to 95°C for 10 minutes followed by 40 cycles of denaturation at 95°C for 10 seconds, annealing at 55°C for 5 seconds, and extension at 72°C for 20 seconds. All PCR reactions were performed in triplicate. Standard curves (cycle threshold values versus template concentration) were prepared for each target gene and for the endogenous reference (*GAPDH*) in each sample. To confirm the specificity of the PCR reaction, PCR products were electrophoresed on a 1.2% agrose gel [3]. Primer sequences are listed in Table 1 [3].

**Table 1:** The sequences of the primers for quantitative RT-PCR.

Gene (Accession No.)	Primer Sequence (5′ to 3′)	Product size (bp)	Tm (°C)
Oct4 (NM_002701)	F: GTGGAGAGCAACTCCGATG R: TGCTCCAGCTTCTCCTTCTC	86	60
Nanog (NM_024865)	F: ATTCAGGACAGCCCTGATTCTTC R: TTTTTGCGACACTCTTCTCTGC	76	60
GAPDH (NM_002046)	F: CATCATCCCTGCCTCTACTG R: GCCTGCTTCACCACCTTC	180	60

### Western blot

The extraction of proteins from cells and western blot analysis were performed as described. Samples (15 µL) were boiled at 95°C for 5 min and separated by 10% SDS-PAGE. The proteins were wet-transferred to Hybond-ECL nitrocellulose paper (Amersham, Arlington Heights, IL, USA). The following primary antibodies were used: rabbit anti–human Oct4, rabbit anti–human Nanog (Santa Cruz Biotechnology, Santa Cruz, CA, USA); rabbit anti-GAPDH (MDBio, Inc., Taipei, Taiwan); and mouse anti–β-actin (Novus Biologicals, Littleton, CO, USA). Immunoreactive protein bands were detected by the ECL detection system (Amersham Biosciences Co., Piscataway, NJ, USA).

### *In vitro* cell invasion Assay

For transwell migration assays, 2×10^5^ cells were plated into the top chamber of a transwell (Corning, Acton, MA, USA) with a porous membrane (8.0 µm pore size). Cells were plated in medium with lower serum (0.5% FBS), and medium supplemented with higher serum (10% FBS) was used as a chemoattractant in the lower chamber. The cells were incubated for 24 hour at 37°C and cells that did not migrate through the pores were removed by a cotton swab. Cells on the lower surface of the membrane were stained with Hoechst 33258 (Sigma-Aldrich Co., St. Louis, MO, USA) to show the nuclei; fluorescence was detected at a magnification of 100x using a fluorescence microscope (Carl Zeiss, Oberkochen, Germany). The number of fluorescent cells in a total of five randomly selected fields was counted. In vitro cell invasion analysis was as described previously [19].

### Soft agar colony forming assay

Each well (35 mm) of a six-well culture dish was coated with 2 ml bottom agar (Sigma-Aldrich Co., St. Louis, MO, USA) mixture (DMEM, 10% (v/v) FCS, 0.6% (w/v) agar). After the bottom layer was solidified, 2 ml top agar-medium mixture (DMEM, 10% (v/v) FCS, 0.3% (w/v) agar) containing 2×10^4^ cells was added, and the dishes were incubated at 37°C for 4 weeks. Plates were stained with 0.005% Crystal Violet then the colonies were counted. The number of total colonies with a diameter ≥100 µm was counted over five fields per well for a total of 15 fields in triplicate experiments [3].

### Statistical analysis

Statistical Package of Social Sciences software (version 13.0) (SPSS, Inc., Chicago, IL) was used for statistical analysis. Student's *t* test was used to determine statistical significance of the differences between experimental groups; *p* values less than 0.05 were considered statistically significant. The level of statistical significance was set at 0.05 for all tests.

## Results

### Inhibition of ALDH1 activity in HNC cells under ALA-PDT treatment

**Figure 1:**
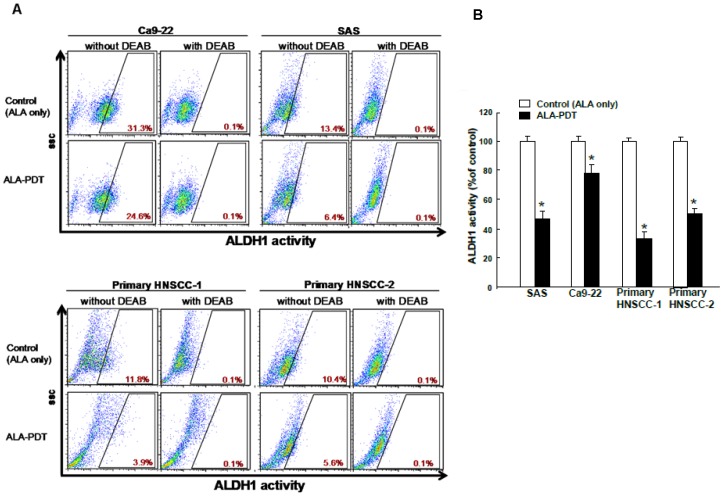
Effects of 5-aminolevulinic acid-mediated photodynamic therapy (ALA-PDT) on ALDH1 activity in HNSCC cell lines and primary HNC cells. (A) HNC cell lines (SAS and Ca9-22) and primary HNC cells (HNC-1 and HNC-2) were seeded as 1×10^6^ cells/well in 6-well-plate and then pretreated 1 mM ALA for 3 hr followed by PDT with red light at a dose of 4 J/cm2 or ALA only treatment control group. ALDH+ cells were determined by Alderfluor assay and viable cells (7-AAD negative) were used for analysis. DEAB-treated cells were used as negative control. (B) The quantification results were shown in bar graph. The experiments were repeated three times and representative results were shown. Results are presented as means ± SD *, p<0.05.

ALDH1, a cytosolic isoenzyme that is responsible for the oxidation of retinol to retinoic acid during early stem cell differentiation have both been shown to be CSC markers in head and neck cancer[20], [21]. First, we used the in vitro cell-based ALDH activity assay system, which has been demonstrated as a HNC-CSCs marker, to examine the effects of ALA-PDT on ALDH1 activity in HNC cell lines and primary cultivated HNC cells by flow cytometry described in material and methods. Our data suggested ALA-PDT treatment significantly decreases ALDH1 activity of HNC cells (Fig. 1).

### ALA-PDT effectively eliminates self-renewal capacity, CD44 positivity, and stemness signatures in HNC-CSCs

**Figure 2:**
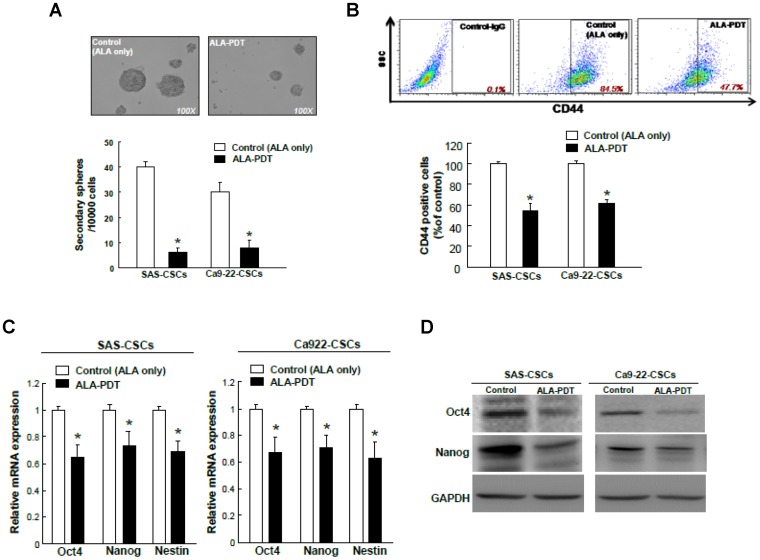
ALA-PDT suppressed secondary sphere-forming capability, CD44 activity, and stemness marker expression in HNC-CSCs. (A) For self-renewal analysis, single cell suspension was obtained from primary HNC-CSCs spheres by accutase digestion and secondary sphere formation capacity was determined with primary sphere culture procedure except the plating cell density as 10000 cells/ml. (B) The expression of CD44 positivity of control and ALA-PDT-treated HNC-CSCs was determined by flow cytometry analysis. Data shown here are the mean ± SD of three independent experiments. *, p<0.05 vs. Control. SAS or Ca9-22-derived HNC-CSCs treated with control or ALA-PDT and analyzed transcripts and protein level of Oct-4 and Nanog by real-time RT-PCR (C) and immunoblotting analysis (D), respectively. *, p<0.05 vs. Control.

Previously, we examined the successful sphere formation over serial passages of culture, one of indexes for evaluating the persistent self-renewal property of cancer stem cells, and showed that the self-renewal capacity of all HNC-CSCs[22]. To investigate the effect of ALA-PDT in maintaining self-renewal HNC-CSCs, we evaluated the secondary sphere-forming ability with ALA-PDT treatment in HNC cells. Treatment of HNC cells with ALA-PDT interfered with spheres body size and numbers of HNC-CSCs (Fig. 2A). Flow cytometry analysis of CD44 expression indicated that the ALA-PDT treatment reduced the percentage of both CD44+ cells in HNC-CSCs (Fig. 2B). To further determine whether the reduction in tumor sphere formation efficiency with ALA-PDT treatment was due to decreased stemness markers expression, stemness genes (Oct-4 and Nanog) of sphere-forming HNC-CSCs with control and ALA-PDT treatment were determined by western blot analysis. The results confirmed that ALA-PDT -treated HNC-CSCs markedly reduced the expression transcript (Fig. 2C) and protein level (Fig. 2D) of stemness genes.

### ALA-PDT treatment delivery enhances the efficacy of chemotherapy in HNC-CSCs

**Figure 3.ALA-PDT:**
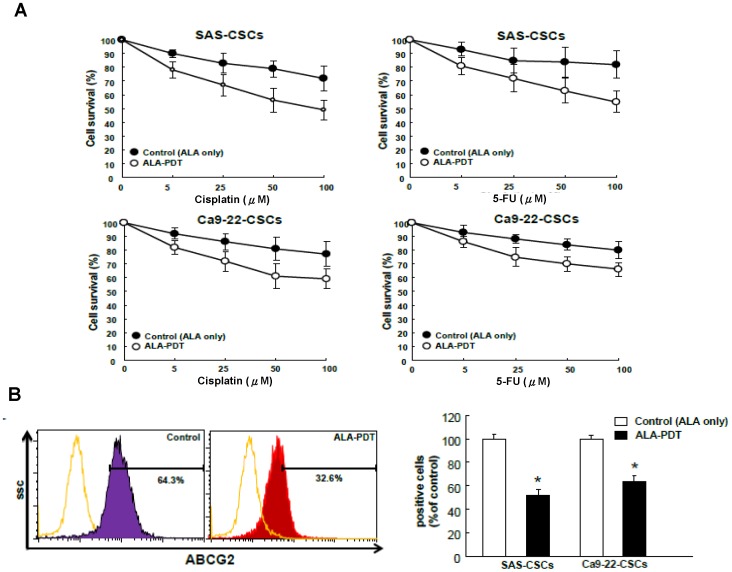
PDT treatment enhances the efficacy of chemotherapy. (A) HNC-CSCs with ALA-PDT or control treatment were subjected to treatment with different concentrations of cisplatin or 5-FU. Cell viability was determined by MTT assay. (B) Flow cytometry analysis of the expression level of drug-resistant marker ABCG2 expression in the HNC-CSCs as indicated.

CSCs are relatively resistant to chemotherapy[6]. The findings that ALA-PDT treatment regulated CSCs properties suggested a role for ALA-PDT as a potential chemo-adjuvant therapy. Cell viability assays showed that the cytotoxic effect on HNC-CSCs to cisplatin and fluorouracil (5-FU) was significantly increased with the ALA-PDT combination treatment (Fig. 3A). Flow cytometry analysis of multidrug resistance (MDR) genes indicated that the decreased chemoresistance by ALA-PDT treatment could be attributed to the reduced expression of ABCG2 (Fig. 3B). These data suggest that ALA-PDT treatment ameliorated the drug resistance of HNC-CSCs to cisplatin and fluorouracil (5-FU) treatment through ABCG2 down-regulation.

### Co-administration of ALA-PDT treatment and cisplatin abrogated invasiveness and clonogenicity of HNC-CSCs

**Figure 4:**
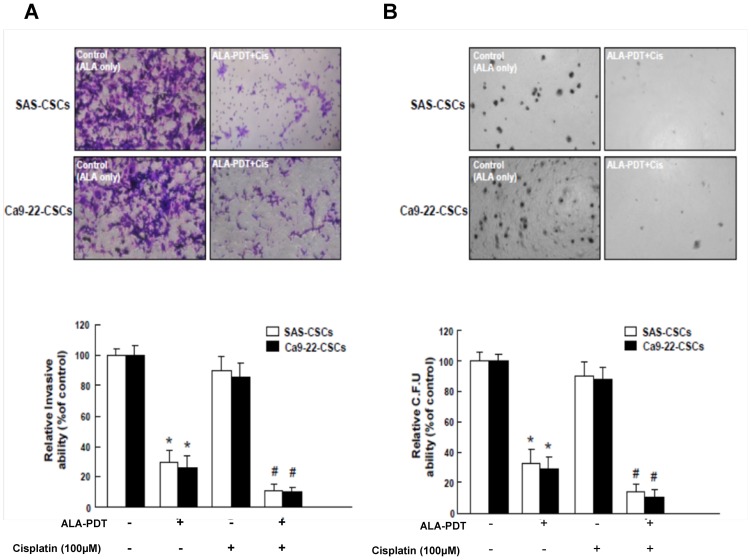
Reduced oncogenic properties in HNC-CSCs by ALA-PDT combined with cisplatin treatment. (A) Invasion ability and (B) colony-forming ability in HNC-CSCs was examined after treatment with either ALA-PDT or cisplatin chemotherapy or both. *, p<0.05 ALA-PDT vs. Control; #, p<0.05 ALA-PDT +Cisplatin vs. ALA-PDT alone.

Since CSCs appear to play a significant role in promoting tumor initiating activity[3], we sought to measure the synergistic effects of ALA-PDT combined with cisplatin treatment on invasion/clonogenicity of HNC-CSCs. Single cell suspension of ALA-PDT-treated HNC-CSCs were treated with or without cisplatin treatment was used for analysis of their invasion/clonogenicity *in vitro* as described in Materials and Methods section. Treatment with cisplatin alone did not affect the invasion ability in HNC-CSCs (Fig. 4A), the combination of ALA-PDT and cisplatin treatment enhanced the efficacy of these treatments (Fig. 4A). Meanwhile, similar synergistic effect of ALA-PDT treatment and chemo-treatment was also observed in colony formation assay (Fig. 4B). The combination treatment showed a synergistic effect in abrogating clonogenicity in HNC-CSCs. These data indicate that the effectiveness of cisplatin chemotherapy treatment on HNC-CSCs can be improved with ALA-PDT treatment.

## Discussion

CSCs are considered to be responsible for the initiation, propagation and metastasis of tumors[23]. Importantly, the existence of CSCs might explain cancer recurrences, even after clinical treatment with either radiotherapy or chemotherapy on cancer patients[24]. Therefore, searching the novel treatment strategy targeting CSCs hopefully provide us with new therapeutic approaches. In the present report, we firstly showed ALA-PDT provided a therapeutic effect in HNC-CSCs by inhibiting the CSCs-like properties of head and neck cancer, such as the stemness signature, migration ability, and chemoresistance. To the best of our knowledge, this is the first study to demonstrate the critical role of an ALA-PDT-based therapy in targeting HNC-derived CSC-like cells and in blocking HNC-CSCs-mediated tumor initiating activity.

Epithelial-mesenchymal transition (EMT) is a process critical for appropriate embryonic development, and it is also re-engaged in adults during tumorigenesis[25]. EMT is widely accepted as one of the CSCs properties, [26] and Oct4/Nanog signaling has been demonstrated to be involved in the regulation of EMT and metastasis [27]. Oral cancer epithelial cells can acquire mesenchymal traits which facilitate migration and invasion through EMT process.[28] It is known that EMT can give rise to cells with stem cell, and cancer initiating stem cells properties that have undergone EMT are therefore more motile and metastasized.[26]. Since we have found that the effect of ALA-PDT on invasion ability in HNC-CSCs, exploring whether the ALA-PDT -mediated CSCs and invasion capabilities depending on EMT pathway will be investigated in the future.

Chemotherapy is the current platform for treating HNC patients with metastasis[29]; however, the chemotherapeutic effect is limited, and its side effects largely interfere with the quality of life of patients. CSCs are clinically characterized by resistance to chemotherapy[29]. The presence of CSCs results in the low efficacy of anti-cancer therapies and the failure of tumor eradication and eventually leads to tumor recurrence and metastasis[30]. The HNC-CSCs were highly resistant to chemotherapy[18], and the chemoresistance of these cells was significantly inhibited when they were treated with ALA-PDT. It is noteworthy that ALA-PDT treatment resulted in reduced MDR protein levels in HNC-derived CSCs after chemotreatment. The detailed mechanisms of the regulatory network between ALA-PDT treatment on drug-resistant genes requires further investigation.

In conclusion, the present study demonstrated the inhibitory effects of ALA-PDT on stem-like properties and chemoresistance in HNC. Notably, here is great need to unravel the underlying mechanisms of the ALA-PDT-mediated pathway in HNC-CSCs and to further evaluate the therapeutic possibilities of ALA-PDT treatment clinically.
